# Characterisation of SARS-CoV-2 genomic variation in response to molnupiravir treatment in the AGILE Phase IIa clinical trial

**DOI:** 10.1038/s41467-022-34839-9

**Published:** 2022-11-26

**Authors:** I’ah Donovan-Banfield, Rebekah Penrice-Randal, Hannah Goldswain, Aleksandra M. Rzeszutek, Jack Pilgrim, Katie Bullock, Geoffrey Saunders, Josh Northey, Xiaofeng Dong, Yan Ryan, Helen Reynolds, Michelle Tetlow, Lauren E. Walker, Richard FitzGerald, Colin Hale, Rebecca Lyon, Christie Woods, Shazaad Ahmad, Dennis Hadjiyiannakis, Jimstan Periselneris, Emma Knox, Calley Middleton, Lara Lavelle-Langham, Victoria Shaw, William Greenhalf, Thomas Edwards, David G. Lalloo, Christopher J. Edwards, Alistair C. Darby, Miles W. Carroll, Gareth Griffiths, Saye H. Khoo, Julian A. Hiscox, Thomas Fletcher

**Affiliations:** 1 Department of Infection Biology and Microbiomes, Institute of Infection, Veterinary and Ecological Sciences, University of Liverpool, Liverpool, UK; 2 NIHR Health Protection Research Unit in Emerging and Zoonotic Infections, Liverpool, UK; 3 Department of Evolution, Ecology and Behaviour, Institute of Infection, Veterinary and Ecological Sciences, University of Liverpool, Liverpool, UK; 4 GCPLab Facility, Institute of Systems, Molecular and Integrative Biology, University of Liverpool, Liverpool, UK; 5 Southampton Clinical Trials Unit, University of Southampton, Southampton, UK; 6 Department of Pharmacology and Therapeutics, Institute of Systems, Molecular and Integrative Biology, University of Liverpool, Liverpool, UK; 7 NIHR Royal Liverpool and Broadgreen Clinical Research Facility, Liverpool University Hospitals NHS Foundation Trust, Liverpool, UK; 8 NIHR Manchester Clinical Research Facility, Manchester University NHS Foundation Trust, Manchester, UK; 9 NIHR Lancashire Clinical Research Facility, Lancashire Teaching Hospitals NHS Foundation Trust, Preston, UK; 10 NIHR Kings Clinical Research Facility, King’s College Hospital NHS Foundation Trust, London, UK; 11 The Clinical Directorate, University of Liverpool, Liverpool, UK; 12 Centre for Drugs and Diagnostics, Liverpool School of Tropical Medicine, Liverpool, UK; 13 Liverpool School of Tropical Medicine, Liverpool, UK; 14 Human Development and Health School, University of Southampton, Southampton, UK; 15 NIHR Southampton Clinical Research Facility, University Hospital Southampton NHS Foundation Trust, Southampton, UK; 16 NIHR Health Protection Research Unit in Gastrointestinal Infections, Liverpool, UK; 17 Wellcome Centre for Human Genetics, Nuffield Department of Medicine, University of Oxford, Oxford, UK; 18 A*STAR Infectious Diseases Laboratories (A*STAR ID Labs), Agency for Science, Technology and Research (A*STAR), Singapore, Singapore

**Keywords:** Antivirals, SARS-CoV-2, Viral genetics, Viral infection

## Abstract

Molnupiravir is an antiviral, currently approved by the UK Medicines and Healthcare products Regulatory Agency (MHRA) for treating at-risk COVID-19 patients, that induces lethal error catastrophe in SARS-CoV-2. How this drug-induced mechanism of action might impact the emergence of resistance mutations is unclear. To investigate this, we used samples from the AGILE Candidate Specific Trial (CST)−2 (clinical trial number NCT04746183). The primary outcomes of AGILE CST-2 were to measure the drug safety and antiviral efficacy of molnupiravir in humans (180 participants randomised 1:1 with placebo). Here, we describe the pre-specified exploratory virological endpoint of CST-2, which was to determine the possible genomic changes in SARS-CoV-2 induced by molnupiravir treatment. We use high-throughput amplicon sequencing and minor variant analysis to characterise viral genomics in each participant whose longitudinal samples (days 1, 3 and 5 post-randomisation) pass the viral genomic quality criteria (*n* = 59 for molnupiravir and *n* = 65 for placebo). Over the course of treatment, no specific mutations were associated with molnupiravir treatment. We find that molnupiravir significantly increased the transition:transversion mutation ratio in SARS-CoV-2, consistent with the model of lethal error catastrophe. This study highlights the utility of examining intra-host virus populations to strengthen the prediction, and surveillance, of potential treatment-emergent adaptations.

## Abstract (web summary)

Molnupiravir is an antiviral that forces lethal error catastrophe in SARS-CoV-2 RNAs. Here, the authors confirm the mechanism of action of molnupiravir in humans using samples obtained from the UK’s AGILE phase IIa clinical trial investigating the antiviral efficacy of the drug against SARS-CoV-2. No treatment-associated SARS-CoV-2 mutations were identified.

## Introduction

The roll-out of oral directly acting antivirals (DAAs) to treat SARS-CoV-2 needs to be accompanied by careful monitoring for potential development of treatment-emergent resistance mutations in current and future circulating variants, as this may limit the public health impact of therapy. DAAs are small molecules which target key stages of the SARS-CoV-2 life cycle. As with human immunodeficiency viruses (HIV), their genetic barrier to resistance will likely differ between drugs, according to their mechanism of action. The activity of DAAs is expected to be less impacted by different SARS-CoV-2 variants compared with monoclonal antibodies, however clinical data are lacking.

Three small molecule DAAs have received early use authorisation for treating COVID-19: remdesivir and molnupiravir (both nucleoside analogues) and nirmatrelvir (which targets the main viral protease). Molnupiravir (β-d-N-hydroxycytidine; NHC) and remdesivir have different modes of administration. The viral mutagen, molnupiravir, can be delivered orally, whereas conventional remdesivir is not orally bioavailable, necessitating its intravenous administration which poses operational barriers to its widespread use. Both remdesivir and molnupiravir are prodrugs, with their active triphosphate metabolites being incorporated by the RNA-dependent RNA-polymerase (RdRp) (NSP12) which is the catalytic core of the replication complex for viral RNA synthesis^1,2^. This encompasses two major processes: (1) replication of the genome involving synthesis of a negative strand template for direct copying of new genomes and (2) discontinuous transcription of sub-genomic messenger RNAs (sgmRNAs). Molnupiravir has a different mechanism of action to remdesivir. The latter directly inhibits the function of the proteins involved in viral RNA synthesis^2^ and the former indirectly interferes with RNA synthesis itself^1,3^.

In human airway cultures and mouse models of disease, molnupiravir (as NHC tri- or mono-phosphate) tautomerizes, leading to the ambiguous binding of either G or A. NHC triphosphate (unbound nucleotide) exhibits a slight preference for binding template G, leading to more G → A than A → G mutations. This same preference also results in more C → U mutations as G → A is the intermediate step for this mutation. This tautomerisation leads to the inhibition of SARS-CoV-2 RNA synthesis by inducing the accumulation of G → A and C → U transition mutations, causing lethal mutagenesis^4^. Molnupiravir seems able to escape proofreading due to the structural stability of the NHC-G and NHC-A base pairs in the RdRp active site, allowing it to avoid triggering backtracking of the RdRp, which is thought to be required for exposing the nascent RNA 3’ end to the exonuclease for excision^5^. The MOVe-OUT phase III double-blinded clinical trial reported that early treatment with molnupiravir reduced the primary efficacy endpoint (incidence of hospitalisation or death at day 29) in at-risk, unvaccinated adults with COVID-19 from 9.7 to 6.8%^6^. Whilst the MOVe-OUT phase III showed promising clinical results, the potential genomic changes of SARS-CoV-2 in response to treatment were not described.

AGILE is the UK early-phase trial platform for the evaluation of SARS-CoV-2 antivirals. AGILE is a partnership between the Southampton Clinical Trials Unit, University of Liverpool, Liverpool School of Tropical Medicine, the NIHR Royal Liverpool and Broadgreen Clinical Research Facility (CRF) and the CRF network^7^. Following the establishment of a recommended phase II dose of molnupiravir^8^, the AGILE CST-2 phase II randomised 180 adult outpatients with confirmed SARS-CoV-2 infection within five days of symptom onset to receive molnupiravir (800 mg twice daily for 5 days) or placebo between the 18^th^ of November 2020 and 16^th^ of March 2022. The primary outcomes of the clinical trial were recently published, and showed that whilst molnupiravir was well-tolerated, the probability that molnupiravir was superior to placebo in reducing time to SARS-CoV-2 PCR negativity was 75.4% - less than the predefined 80% threshold for recommending a candidate drug for large-scale evaluation^9^.

## Results

**Fig. 1:**
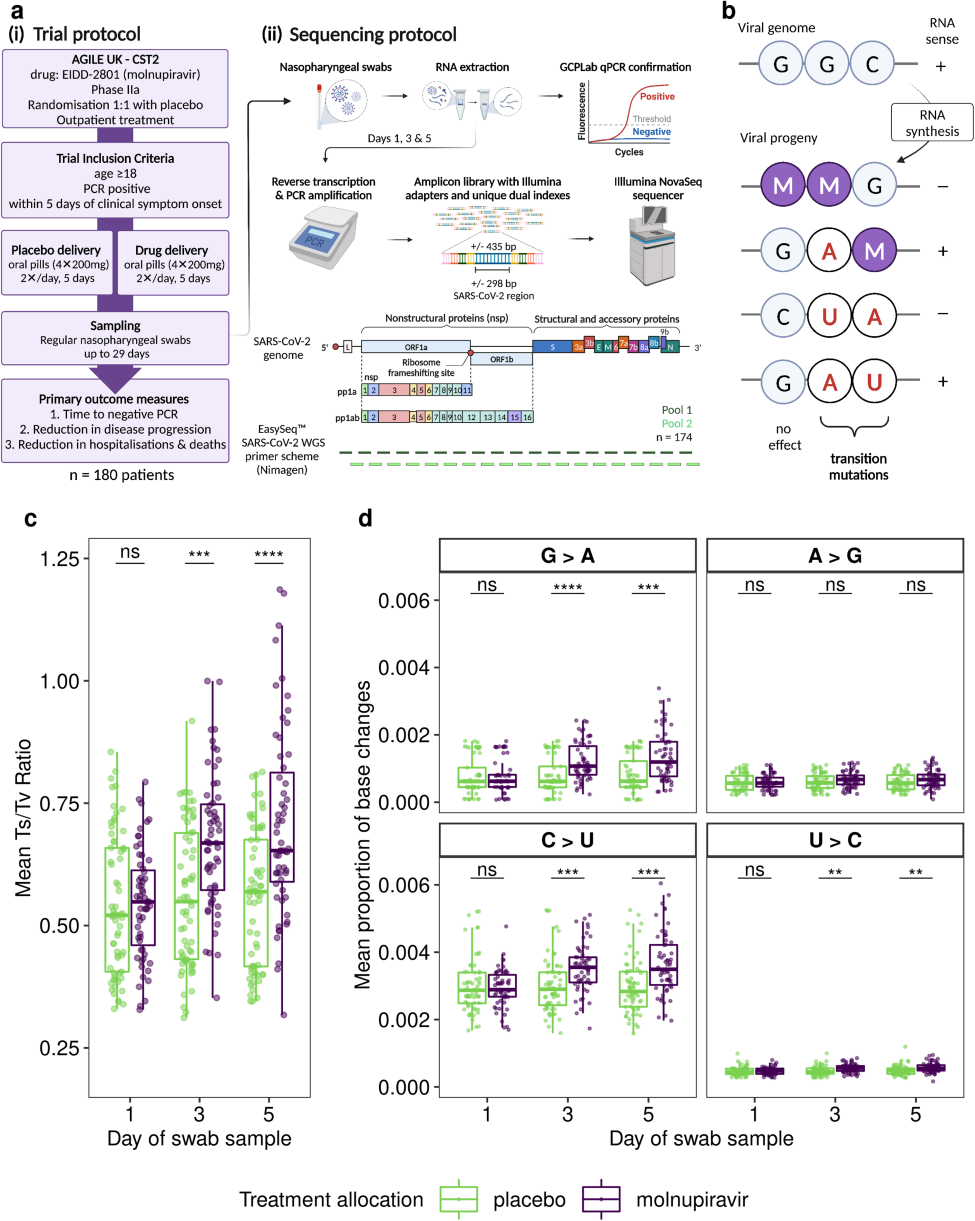
Protocol overview and the detection of the molecular signatures of molnupiravir mechanism of action. **a** (i) A simplified AGILE CST-2 Phase IIa trial protocol. Molnupiravir was administered to outpatients as four oral pills (200 mg each, 800 mg total) every 12 hours for five days. Participants were randomised placebo to drug 1:1, with nasopharyngeal swabs taken for viral load monitoring. (ii) Sequencing protocol. RNA extracted from nasopharyngeal swabs, taken at days 1, 3 and 5 post treatment initiation, was used for amplicon library preparation using the EasySeq™ RC-PCR SARS-CoV-2 WGS kit (Nimagen, Netherlands). Resulting sequence reads were mapped to the Wuhan-Hu-1 reference (NC_045512.2). **b** Molnupiravir mechanism of action via the RNA template leads to the accumulation of transition mutations in viral progeny. **c** Average Ts/Tv ratio values per RNA sample from all participants (placebo *n* = 65, green; molnupiravir *n* = 59, purple). SARS-CoV-2 RNA from molnupiravir (purple) participants show a statistically significant accumulation of transition mutations over time compared to placebo (green). **d** the same genomic data as in c separated into the individual base changes that contribute to the transition mutation counts. A two-sided Wilcoxon rank sum test was performed in **c** and **d**; ****P ≤ 0.0001, ***P ≤ 0.001, **P ≤ 0.01, ns = *P* > 0.05 (Bonferroni adjusted). The boxplots indicate the median, interquartile range, and the minimum and maximum values (excluding outliers). Exact *p* values are reported in the Source Data. RC-PCR reverse complement-polymerase chain reaction, WGS whole genome sequencing, GCPLab good clinical practice laboratory (University of Liverpool). Fig. **a** and **b** were created with Biorender.com.

To investigate the exploratory endpoint of AGILE CST-2, serial nasopharyngeal samples from all 180 patients (taken at days 1, 3 and 5 post treatment initiation) were sequenced to investigate potential drug-induced viral adaptation and confirm the mechanism of action of molnupiravir (Fig. 1a(i)). An amplicon-based deep sequencing approach was used to determine the SARS-CoV-2 genome to high sequence read depth such that both lineage assignment of the dominant genome sequence and minor genomic variant information could be generated to enable identification of the mechanism of action (Table 1, Fig. 1a(ii)). Participants were included in the minor variant analysis if all three of their samples met the following criteria: 1) the dominant genome sequence had a minimum 90% consensus called and 2) 90% of genome positions had a minimum coverage of 200X. Using these criteria, longitudinal samples from 65 participants receiving placebo and 59 participants treated with molnupiravir were identified for SARS-CoV-2 genomic analysis. Stringent genome quality criteria were used to ensure that longitudinal samples from each patient had comparable genome quality but resulted in the exclusion of approximately 32% of all samples from the final analysis (35% of molnupiravir and 28% of placebo samples; Table 1). Most of the samples that failed to meet the criteria were Day 3 or 5 samples with Ct values ≥ 28 (low viral load). A decrease in viral load (resulting in an increase in Ct value) is the natural trajectory of an acute infection, however it can make balanced whole genome comparisons difficult. The genome selection criteria allowed for the avoidance of having to accommodate regions of low or no sequence coverage which can skew estimates of within-host diversity^10^.

**Table 1:** Lineage assignment of SARS-CoV-2 from participants enrolled in the AGILE CST-2 phase IIa molnupiravir clinical trial

Lineage	Placebo	Molnupiravir
	Total (passed)	Total (passed)
B.1.1.7 (Alpha)	20 (14)	17 (11)
B.1.1.1	1 (1)	0 (0)
B.1.177 (EU1)	13 (10)	15 (8)
Delta (all)	35 (24)	37 (28)
B.1.617.2	2 (0)	2 (2)
AY.120	1 (1)	0 (0)
AY.33	0 (0)	1 (1)
AY.4	28 (21)	30 (22)
AY.43	0 (0)	1 (1)
AY.4.2	2 (1)	2 (2)
AY.4.2.1	1 (1)	0 (0)
AY.98	1 (0)	1 (1)
Omicron (all)	19 (16)	20 (12)
BA.1	12 (9)	15 (11)
BA.2	6 (6)	5 (1)
XE	1 (1)	0 (0)
Failed to assign	2 (0)	1 (0)
Trial total	90 (65)	90 (59)

Viral RNA from nasopharyngeal swabs obtained from participants enrolled in the phase IIa clinical trial was sequenced as described in Methods. The consensus SARS-CoV-2 genome for each sample, assembled after mapping to the Wuhan-Hu-1 reference genome, was used to assign the lineage of SARS-CoV-2 that each patient was infected with upon entering the trial, using the software tool, Pangolin (version 4.0.6). Only participants that passed criteria of all samples (Days 1, 3 and 5) with a minimum 90% genome coverage at 200X were included in downstream analyses – numbers indicated in brackets for each (sub-)lineage. Sub-lineages denoted in italics. Lineages that only had one patient or an uneven balance of placebo:drug were excluded from the analysis (B.1.1.1. and BA.2).

Molnupiravir was predicted to increase the number of mutations in the genome of SARS-CoV-2 (Fig. 1b) and that this would manifest as an increase in the transition/transversion (Ts/Tv) ratio^11^. The sequencing data indicated that transition mutations were significantly increased in viral RNA from molnupiravir treated participants at Day 3 or Day 5 compared to participants given a placebo (Fig. 1c). The frequency of C → U mutations were higher than those for G → A (Fig. 1d). U → C mutations were also significantly increased. All other base changes showed no increase over time in either group (Supplementary Fig. 1).

**Fig. 2:**
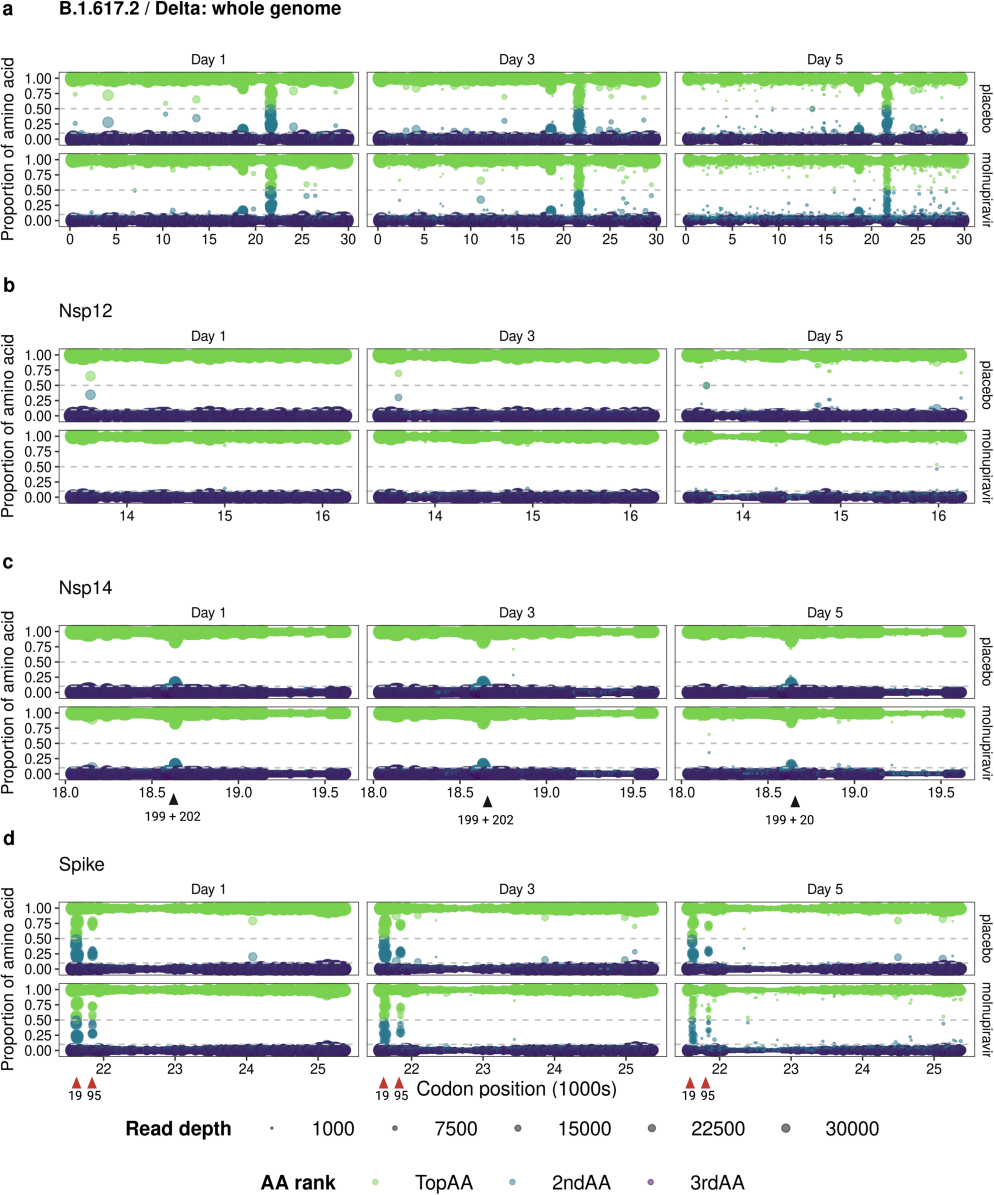
Predicted amino acid variations derived from SARS-CoV-2 RNA in the whole genome, NSP12, NSP14 and Spike sequences. **a** Predicted amino acid variation derived from RNA sequence information across the whole genome in all Delta infected participants (*n* = 52). Each sample is assigned a predicted “Top” (green), “2nd” (blue) and “3rd” (dark purple) amino acid (AA) based on proportion of reads at every genome position. Minimum read depth = 200. Minor genomic variants (>0.1 and <0.5; grey dashed lines) increase in frequency over time, with viral RNA from molnupiravir treated participants showing more diversity. **b** NSP12 showed very little minor genomic variation over the five days. **c** NSP14 also showed minor genomic stability, but had sites of low-level minor variation at codon positions 18,634 and 18,643 (indicated as amino acids 199 and 202 with black arrows) that were present in all samples tested and may represent a persistent sub-population. **d** Spike had two sites with an amino acid mixed population at codon positions 21,617 and 21,845 (indicated as amino acids 19 and 95 with red arrows) in all Delta samples analysed. These are known VOC sites in all the Delta sub-lineages.

The implications of greater viral diversity in response to molnupiravir treatment are currently unknown, but it could potentially influence the genetic barrier to resistance. To address this, SARS-CoV-2 sequence was translated in silico at both the dominant and minor variant genome level and treatment-emergent mutations were analysed to assess preferential enrichment of mutations (i.e., was there a greater chance of mutations arising during treatment and then persisting in these regions thereafter). Given the mechanism by which molnupiravir may avoid detection by viral replication machinery, we postulated that the two coding regions more likely to be subject to selection pressure would be *nsp12* (the RNA dependent RNA polymerase; RdRp) and *nsp14* (the exonuclease). Being able to detect the incorporation of molnupiravir instead of natural nucleotides in the nascent RNA would allow either NSP12 or NSP14 to stall or back-track and excise the mis-incorporated nucleotide analogue. Two previous in vitro studies on the incorporation of molnupiravir into the nascent RNA strand found that molnupiravir did not cause polymerase stalling, but one of the studies demonstrated that molnupiravir was capable of inducing chain termination^1,3^. If chain termination occurred, this may have placed selection pressure on both the RdRp and the exonuclease to counter the effects of molnupiravir. This is similar to what has recently been described for SARS-CoV-2 in an immunocompromised patient treated with remdesivir^12^. In our study, the data indicated that there was no change in the predicted amino acid sequence of NSP12 and NSP14 at the dominant genome level over the five days of molnupiravir treatment (Fig. 2b, c).

**Fig. 3:**
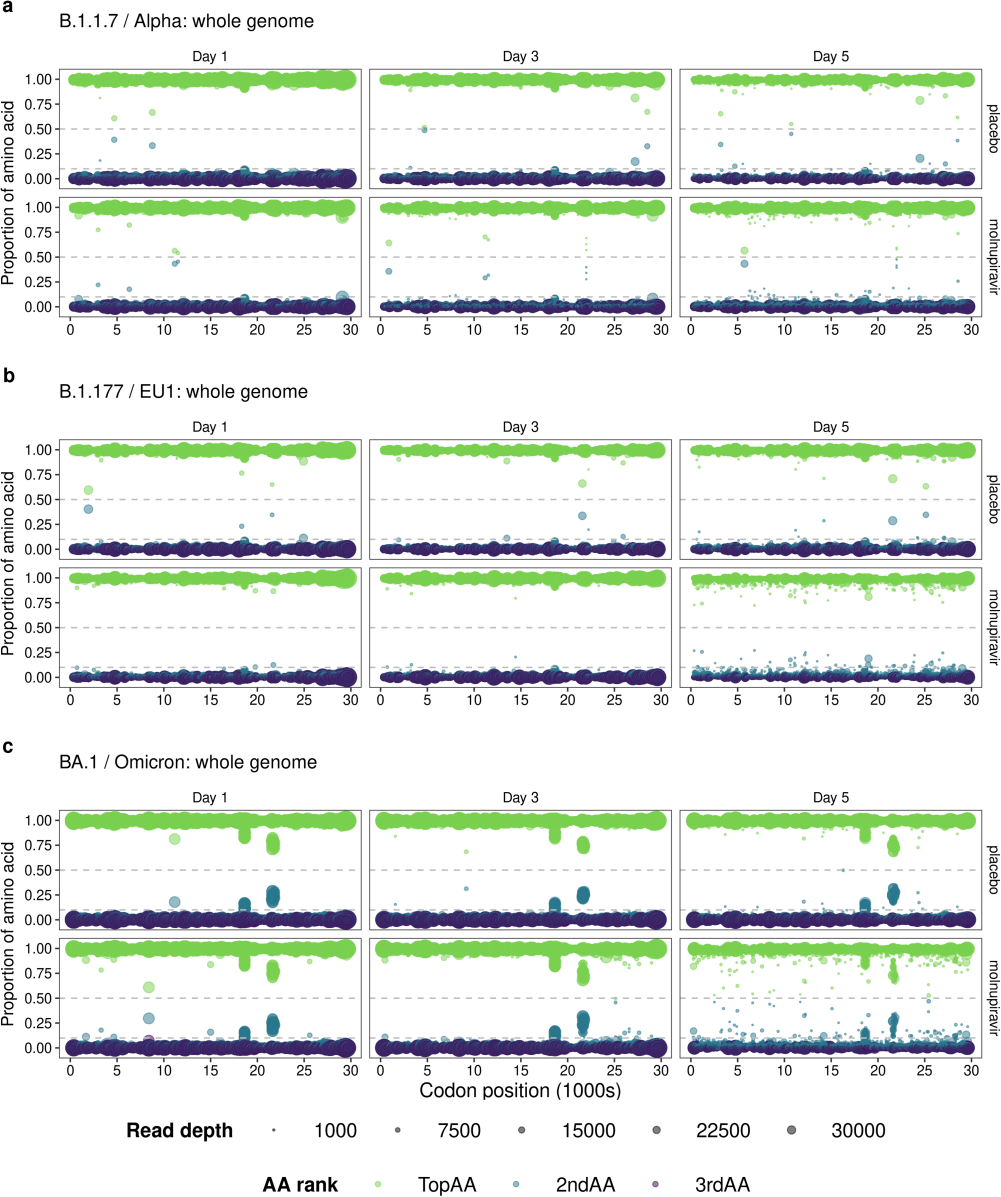
Predicted amino acid variations derived from SARS-CoV-2 RNA in the whole genome of B.1.1.7/Alpha, B.1.177/EU1 and BA.1/Omicron lineages. Predicted amino acid variation derived from RNA sequence information across the whole genome in all **a**, alpha (placebo *n* = 14, molnupiravir *n* = 11); **b** B.1.177/EU1 (placebo *n* = 10, molnupiravir *n* = 8); and **c** BA.1/Omicron (placebo *n* = 9, molnupiravir *n* = 11) infected participants. Each sample is assigned a predicted “Top” (green), “2nd” (blue) and “3rd” (dark purple) amino acid (AA) based on proportion of reads at every genome position. Minimum read depth = 200. Minor genomic variants (>0.1 and <0.5; grey dashed lines) increase in frequency over time, with viral RNA from molnupiravir treated participants showing more diversity.

Reflecting the change in the Ts/Tv ratio, the diversity of the predicted amino acid sequence increased over the course of infection in both treatment groups. The spread of diversity was reflected across the genome, with a slight bias towards the 3′ end. More diversity was observed in the Day 5 samples from the molnupiravir-treated group compared to the placebo group (Fig. 2 - with data from participants infected with the Delta variant of concern (VoC) viruses as an example). A similar pattern was found in participants infected with other VoCs (Fig. 3). Curiously, two positions in NSP14 had a slightly increased diversity (codon positions 18634 and 18643; NSP14 amino acid positions 199 and 202) that were present in samples from both treated and placebo groups and may represent a persistent sub-population (Fig. 2b and Supplementary Figs. 2b, 3b and 4b).

To understand any risks of combining molnupiravir with monoclonal antibody treatment, we also focused on amino acid substitutions in the spike protein. Two of the codons starting at positions 21,617 and 21,845 (amino acid 19 and 95, respectively), which are known lineage-defining mutation sites in all Delta sub-lineages, were variable in participants from both treated and placebo control groups (Fig. 2d). Similarly, codons starting at positions 21,620 and 21,638 in the BA.1 spike gene (amino acids 20 and 26 respectively) showed increased diversity, regardless of treatment group or visit day (Fig. 3c and Supplementary Fig. 4c). These positions are in the N-terminal domain of the spike protein, with mixed populations of T20N and P26S amino acid substitutions. A search was conducted on outbreak.info^13^ (a platform that tracks mutations using the GISAID^14^ SARS-CoV-2 sequence database) to see whether the minor variant substitutions (S:T20N and S:P26S) were reported in global SARS-CoV-2 dominant genome (consensus-level) sequences. Both were present with a frequency less than 0.5% and 1% of worldwide sequences, respectively. It is possible that these sites widely exist with persistent minor genome variations, but this minor variant level information is not reported in sequence repositories that only publish dominant genome level variations (frequency of > 50%). Both the BA.1 and Delta lineages displayed higher predicted amino acid diversity across the genome at Day 5 than the other SARS-CoV-2 lineage (Figs. 2a and 3d and Supplementary Figs. 2a, 3a and 4a). This could be because they are the most divergent lineages from the Wuhan-Hu-1 reference genome that the sequence reads are mapped to, in combination with the fact both lineages have several sub-lineages as detailed in Table 1.

To our knowledge, this is the first confirmation of the mechanism of molnupiravir on viral replication in humans infected with SARS-CoV-2, following the currently approved dosing regimen, in the UK. In the molnupiravir treated group, the Ts/Tv mutation ratio was higher than in the placebo group. This corresponded with higher C → U and G → A mutations than other combinations. The increase in this ratio corresponded to the length of treatment, with the greatest diversity seen on Day 5. There were no amino acid substitutions in SARS-CoV-2 that were enriched consistently at specific sites in the molnupiravir-treated group at any of the sampled times, including in the coding regions for NSP12 and NSP14.

## Discussion

During acute SARS-CoV-2 infection, viral mutations that are either neutral, detrimental, or beneficial can occur. Treatment with molnupiravir aims to surmount the threshold of tolerated detrimental mutations (leading to lethal error catastrophe), such that viral replication is diminished, resulting in a concomitant reduction in viral load. This study revealed the intricacies of this mechanism of action in humans infected with SARS-CoV-2. This study also highlighted the utility of minor genomic variant analysis in examining intra-host virus populations which strengthens the prediction, and surveillance, of treatment-emergent adaptations. A deep-sequencing and bioinformatic pipeline for handling and visualising minor variant data was established and can be used with other antiviral treatments for SARS-CoV-2 or similar viral infections. In future, such approaches can be used by regulatory bodies and public health officials to inform approval decisions and surveillance of resistance in the wake of large-scale administration of newly approved drugs. The data described complements the clinical findings of AGILE CST-2 and has provided comprehensive information regarding drug effects on viral genomes. However, it is important to highlight that this study does not seek to comment on whether the development of molnupiravir resistance is possible. This was a controlled clinical trial, with enrolled participants adhering to the dosing regimen and subject to close monitoring. This is unlikely to happen in real-life contexts and thus selection pressures and opportunities for onward transmission are not the same. We would caution that this data should not be used as evidence of virological safety, but instead act as a foundation for further investigations. The only way this can be comprehensively achieved is for widespread virological surveillance that accompanies the roll-out of a therapeutic at scale in the general population, monitoring closely for accumulated mutations that might point to resistance mechanisms.

## Methods

### Sample collection

AGILE is a randomised multi-arm, multi-dose, phase I/IIa platform in the UK using a seamless Bayesian adaptive design to determine the safety, activity, and optimal dose of multiple SARS-CoV-2 candidate therapeutics^7^. This trial evaluated molnupiravir (EIDD-2801/MK-4482), for the treatment of COVID-19 in a seamless phase I/II trial (clinicaltrials.gov registration number NCT04746183). Using a permuted block (block size 2 or 4) method and stratifying by site, participants were randomly assigned (1:1) to receive either molnupiravir plus standard of care or placebo plus standard of care. The randomisation sequence was generated by use of STATA (version 16) by an independent statistician (who had no further involvement in the trial) and used to prepare labelled placebo and treatment packs, which were assigned sequentially to patients on randomisation. Placebo and molnupiravir were provided in tablets of identical appearance. Eligible participants were men and women aged ≥18 years with PCR-confirmed SARS-CoV-2 infection who were within five days of symptom onset, free of uncontrolled chronic conditions, and ambulant in the community with mild or moderate disease. All participants provided written, informed consent before enrolment. Nasopharyngeal swabs were obtained from participants on days 1, 3, 5, 8, 11, 15, 22 and 29. Only samples taken on days 1, 3 and 5 were sequenced for this analysis. The full study protocol can be found in the supplementary information of the paper detailing the clinical findings of AGILE CST-2^9^. Participants’ co-variate information is available in the Source Data.

### RNA extraction, amplicon library preparation and Illumina sequencing

RNA was extracted from the nasopharyngeal swabs by the GCP Laboratory Facility at the University of Liverpool using a Maxwell® RSC instrument, an automated nucleic acid extraction instrument (Promega, USA). Aliquots of surplus RNA were provided for sequencing analysis. For each participant, there were three samples (from Days 1, 3 and 5), all sequenced once. Briefly, library preparation consisted of converting RNA to cDNA using LunaScript™ (Thermofisher, Waltham, Massachusetts), then amplified by reverse complement (RC)-PCR amplification (EasySeq™ SARS-CoV-2 Whole Genome Sequencing Kit (V3) RC-COV096, NimaGen, Nijmegen, The Netherlands)^15^. This kit barcodes and ligates Illumina adapters in a single PCR reaction, with two separate pools of primers (pools 1 and 2). After amplification, primer pools 1 and 2 for each amplified sample were mixed 1:1 before being cleaned with Beckman Coulter™ Agencourt AmpureXP beads (Fisher Scientific, Hampton, New Hampshire), quantified and the library quality assessed on an Agilent 2100 Bioanalyzer (Agilent, Santa Clara, California). All purified samples were then pooled together and denatured. Finally, the denatured amplicon library was loaded into the NovaSeq cartridge (2 × 150 bp run) before loading on the NovaSeq 6000 machine. The sequencing was conducted in two separate sequencing runs, one for the first 120 participants’ swab samples, and a second for the final 60 participants’ swab samples.

### In silico processing

The raw sequencing data was processed using two different pipelines (summarised in Supplementary Fig. 5). The first method, EasySeq_covid19 (version 0.9, code available at https://github.com/JordyCoolen/easyseq_covid19), performs quality control steps, maps to the reference genome (Wuhan-Hu-1; NC045512.2), variant calls and generates a consensus genome for each sample^15^. Default parameters were used and are as follows: variant call threshold = 0.5; variant calling quality threshold = 20; variant calling minimum depth = 10. Pangolin (version 4.0.6) was used to assign SARS-CoV-2 lineage, with maximum ambiguity set at 0.3^16^. The second method, DiversiTools (code available at https://github.com/josephhughes/DiversiTools), uses the primer-trimmed alignment file (named as [sampleID]_L001.final.bam) and its associated index file (produced in the EasySeq pipeline) along with the reference genome and a coding region file to analyse the minor genomic variation and predict the amino acid sequence based on the genomic data. DiversiTools allows for an in-depth analysis of viral diversity in each sample, rather than just the consensus/dominant genomic information, as previously described^17^. Participants were included in the minor variant analysis if all three of their samples met the following criteria: 1) the dominant genome sequence had a minimum 90% consensus called and 2) 90% of genome positions had a minimum coverage of 200X. Data visualisation was conducted in R (version 4.0.2), using the tidyverse package (version 1.3.2) for data manipulation. Wilcoxon rank sum tests (two-sided) were used to determine differences between treatment groups at each time point, reporting the *p*-adjusted value (Bonferroni), using the Rstatix package (version 0.7.0). All plots were created using ggplot2 (version 3.3.6). Figures were compiled using cowplot (version 1.1.1) and magick (version 2.7.3) packages. Schematic Fig. 1a, b and Supplementary Fig. 5 were created using Biorender.com.

### Reporting summary

Further information on research design is available in the Nature Portfolio Reporting Summary linked to this article.

## Supplementary information

Supplementary Information

Peer Review File

Reporting Summary

## Data Availability

All raw sequencing data used in the analysis have been deposited to the National Center for Biotechnology Information (NCBI) Short Read Archive (SRA) under accession code PRJNA854613. Source data has been included and details 1. Genomic analysis metadata; 2. The exact *p-*values for Fig. 1c, 1d and Supplementary Fig. 1; 3. Accession numbers for all the raw sequence files deposited in the SRA repository PRJNA854613; and 4. The trial participants’ co-variate information. Source data are provided with this paper.

## Source data

Source Data

## Code availability

All custom code used in this study is available at https://github.com/Hiscox-lab/AGILE-molnupiravir-viral-genomics. A citeable repository is available at Zenodo.org, 10.5281/zenodo.7245793^18^.
