# Supplementary material for: Characterisation of SARS-CoV-2 genomic variation in response to molnupiravir treatment in the AGILE Phase IIa clinical trial
Source: Nat Commun. 2022 Nov 26;13:7284. doi: 10.1038/s41467-022-34839-9 (PMC9701236; doi:10.1038/s41467-022-34839-9)
Supplement: Supplementary file 1 — Supplementary Information [file 41467_2022_34839_MOESM1_ESM.pdf]

Supplementary Information

Characterisation of SARS-CoV-2 genomic variation in response to molnupiravir treatment in the AGILE Phase IIa clinical trial.

Donovan-Banfield *et al.*, 2022.

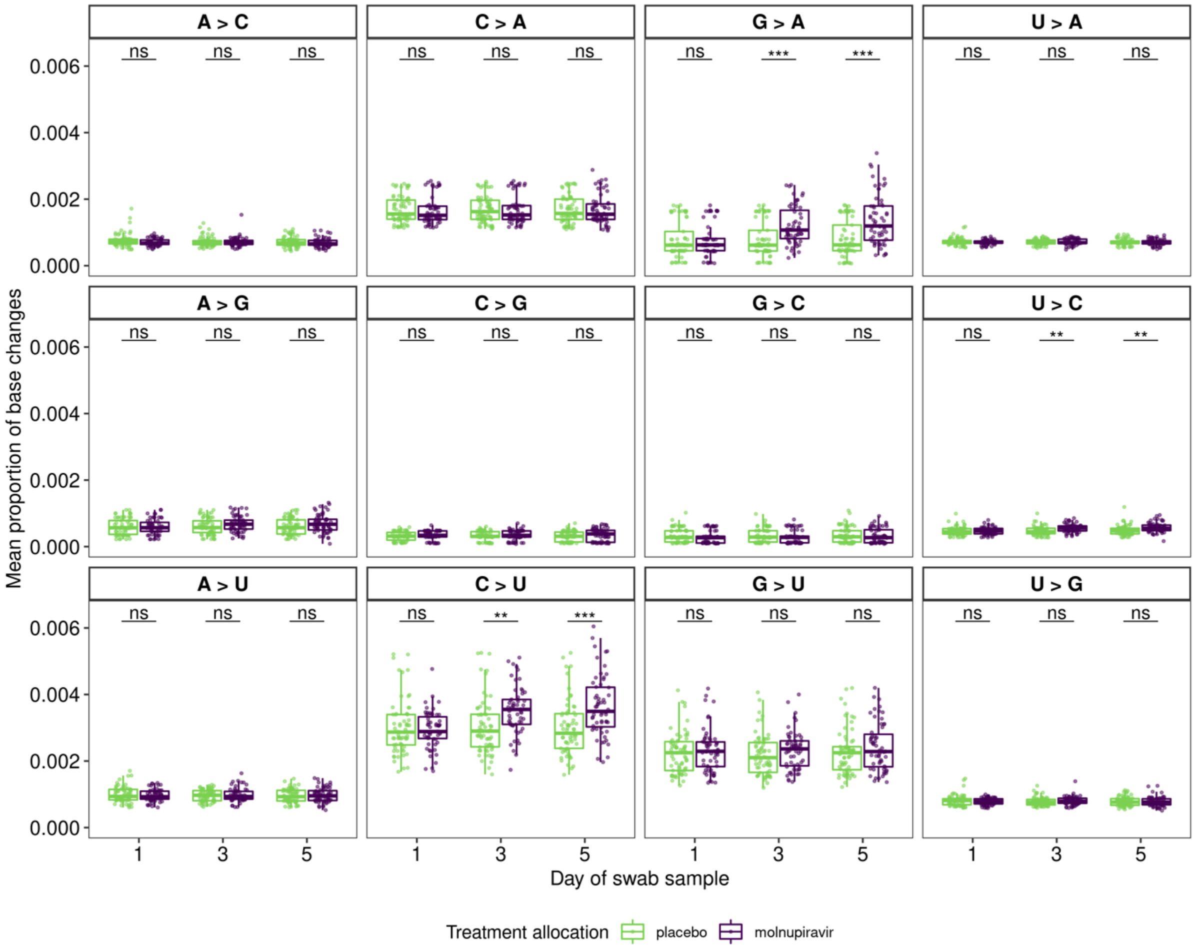

Supplementary Fig. 1: All base changes over time. The mean frequency of all possible base change combinations was calculated per sample, with data grouped by treatment (placebo n = 65, green; molnupiravir n = 59, blue) and day of swab sample. A two-sided Wilcoxon rank sum test was performed, to calculate the statistical significance of the mean difference in bases change frequency between treatment groups on each sample day. Of the twelve possible base changes, only 'G > A', 'C > U' and 'U > C' showed statistically different mean frequencies between groups at Days 3 and 5. *****P* ≤ 0.0001, ****P* ≤ 0.001, ***P* ≤ 0.01, ns = *P* > 0.05 (Bonferroni adjusted). The boxplots indicate the median, interquartile range, and the minimum and maximum values (excluding outliers). Exact *p* values are reported in the Source Data.

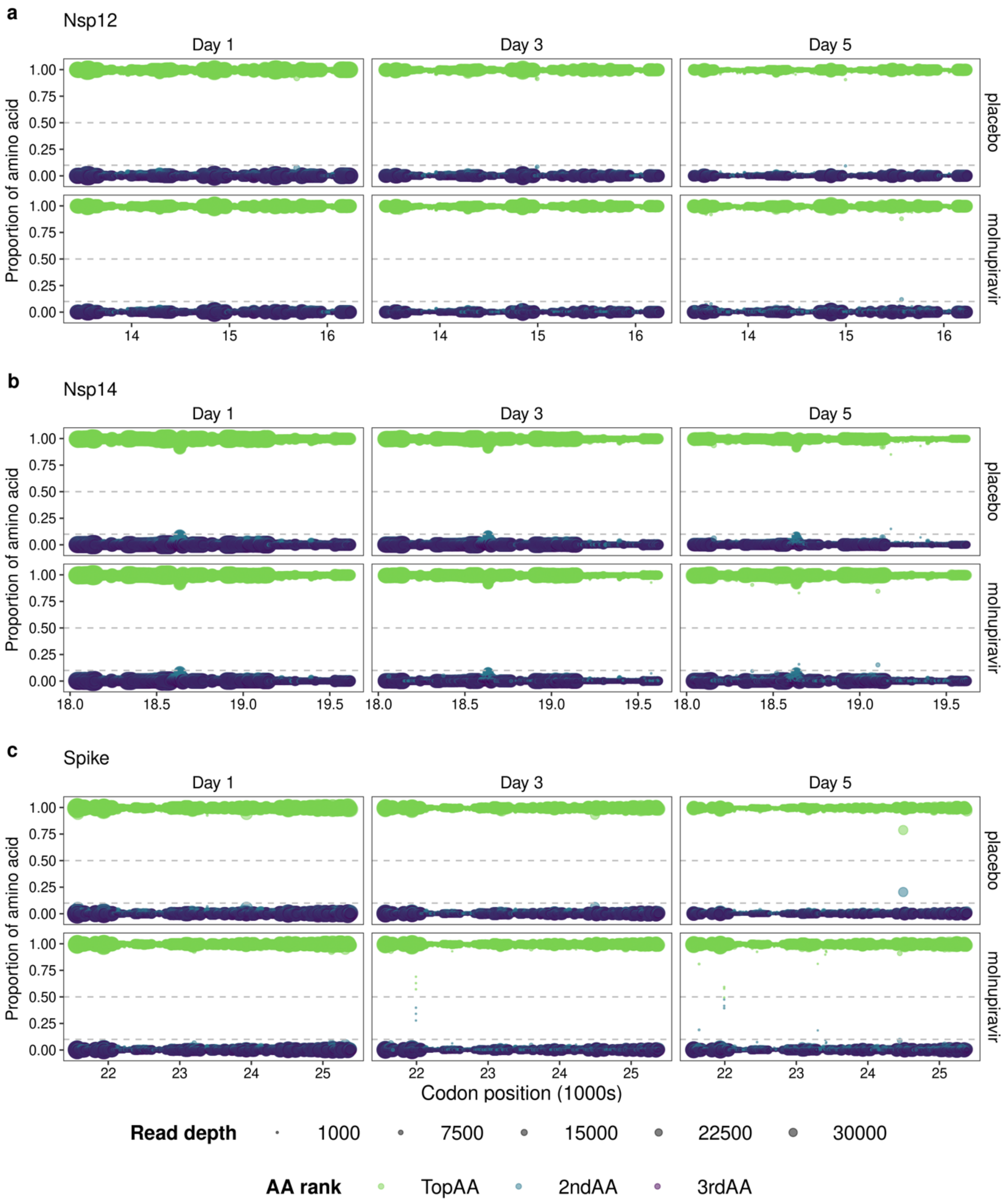

Supplementary Fig. 2. B.1.1.7/Alpha - predicted amino acid variations in Nsp12, Nsp14 and Spike proteins. Alpha variation in **a**, NSP12; **b**, NSP14; and **c**, Spike (placebo n=14, molnupiravir n=11). Each sample is assigned a predicted "Top" (green), "2nd" (blue) and "3rd" (dark purple) amino acid (AA) based on proportion of reads at every genome position. Minimum read depth = 200. Minor genomic variants (>0.1 and <0.5; grey dashed lines) increase in frequency over time, with viral RNA from molnupiravir treated participants showing more diversity.

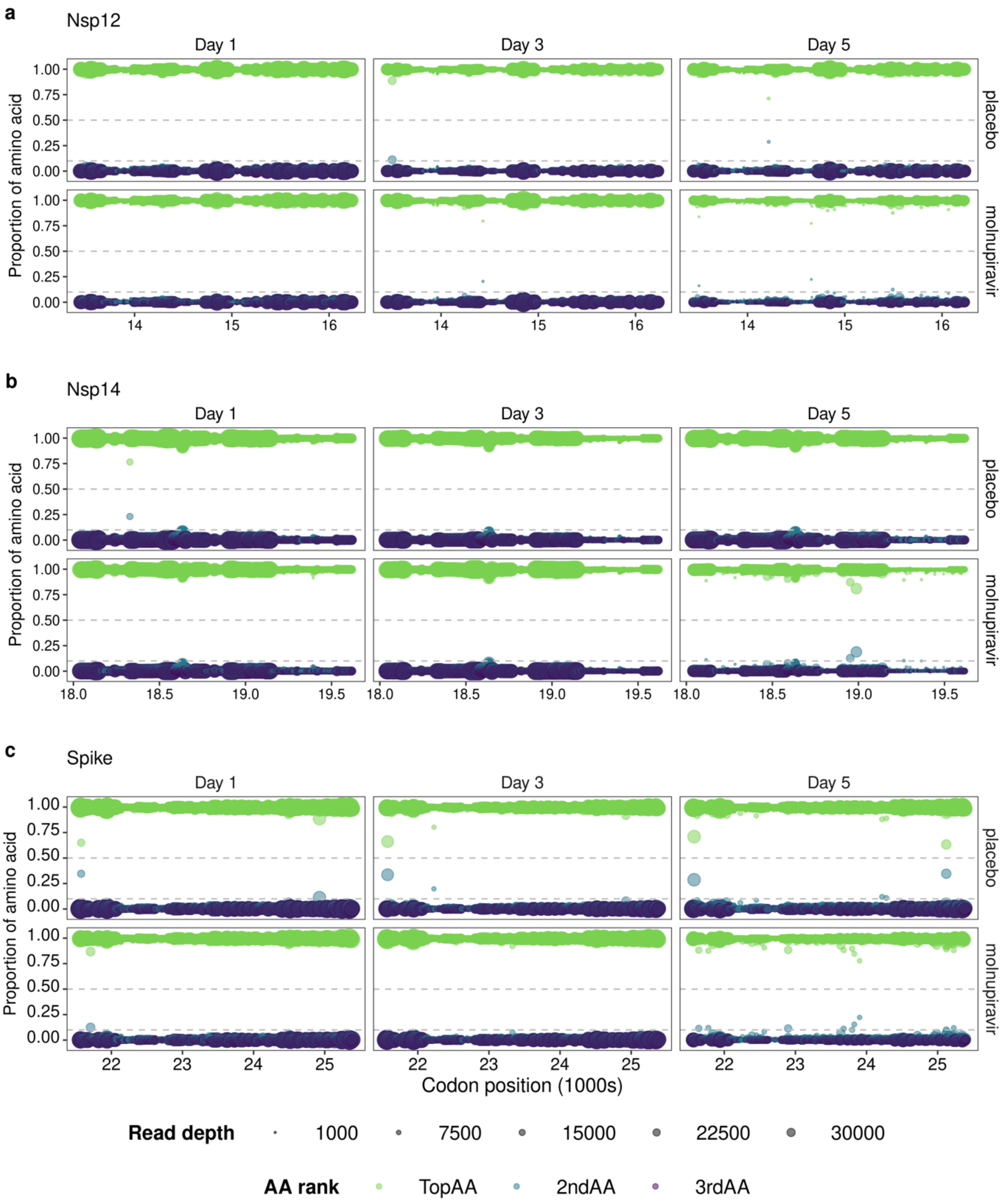

Supplementary Fig. 3. B.1.177/EU1 - predicted amino acid variations in Nsp12, Nsp14 and Spike proteins.

EU1 variation in **a**, NSP12; **b**, NSP14; and **c**, Spike (placebo n=10, molnupiravir n=8). Each sample is assigned a predicted "Top" (green), "2nd" (blue) and "3rd" (dark purple) amino acid (AA) based on proportion of reads at every genome position. Minimum read depth = 200. Minor genomic variants (>0.1 and <0.5; grey dashed lines) increase in frequency over time, with viral RNA from molnupiravir treated participants showing more diversity.

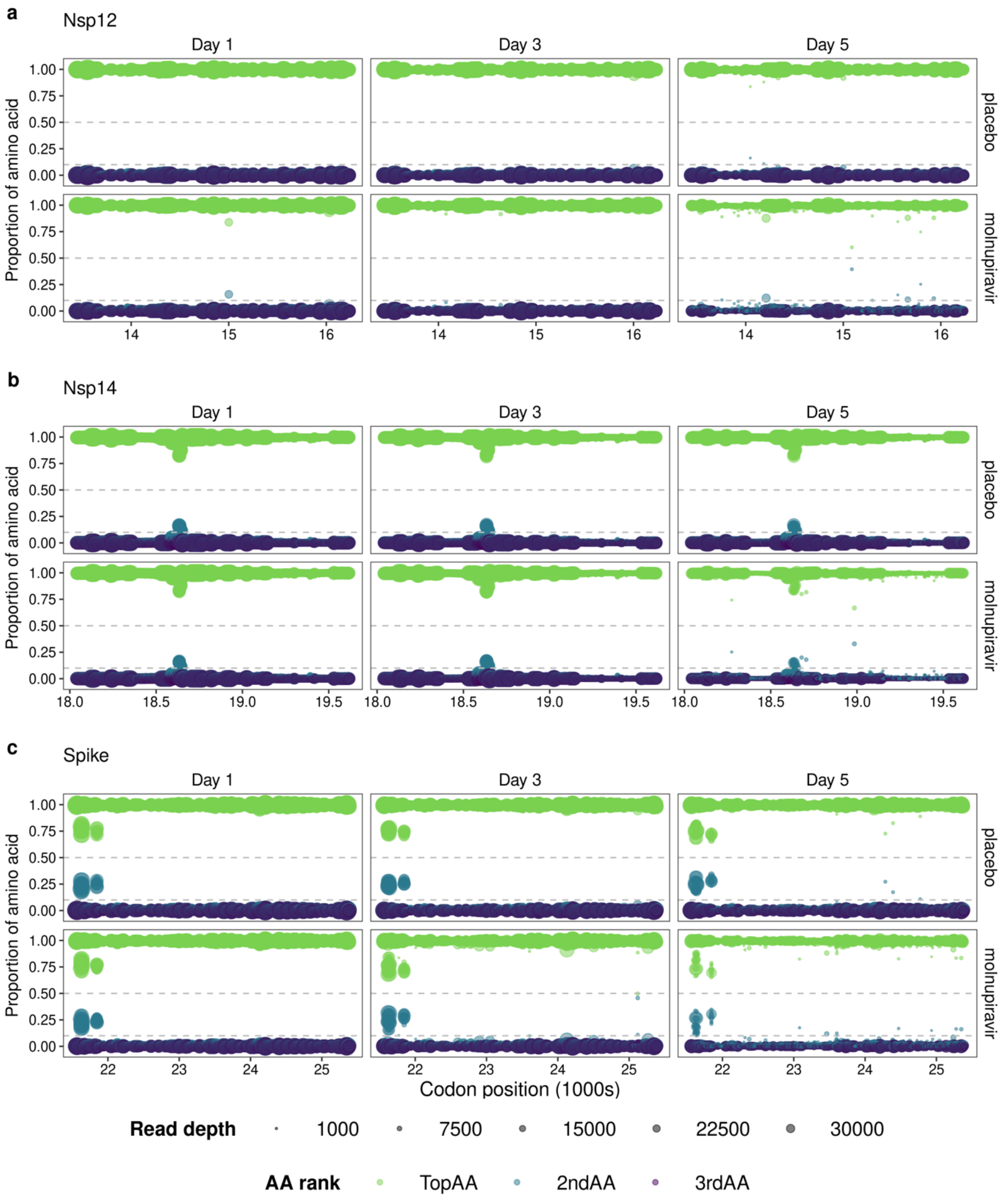

Supplementary Fig. 4. BA.1/Omicron - predicted amino acid variations in Nsp12, Nsp14 and Spike proteins.

BA.1 variation in **a**, NSP12; **b**, NSP14; and **c**, Spike (placebo n=9, molnupiravir n=11). Each sample is assigned a predicted “Top” (green), “2nd” (blue) and “3rd” (dark purple) amino acid (AA) based on proportion of reads at every genome position. Minimum read depth = 200. Minor genomic variants (>0.1 and <0.5; grey dashed lines) increase in frequency over time, with viral RNA from molnupiravir treated participants showing more diversity. Persistent sub-populations identified in Spike (**c**) at codon positions 21620 and 21638 for all BA.1 infected participants, indicated by red arrows and labelled as amino acid positions 20 and 26 respectively.

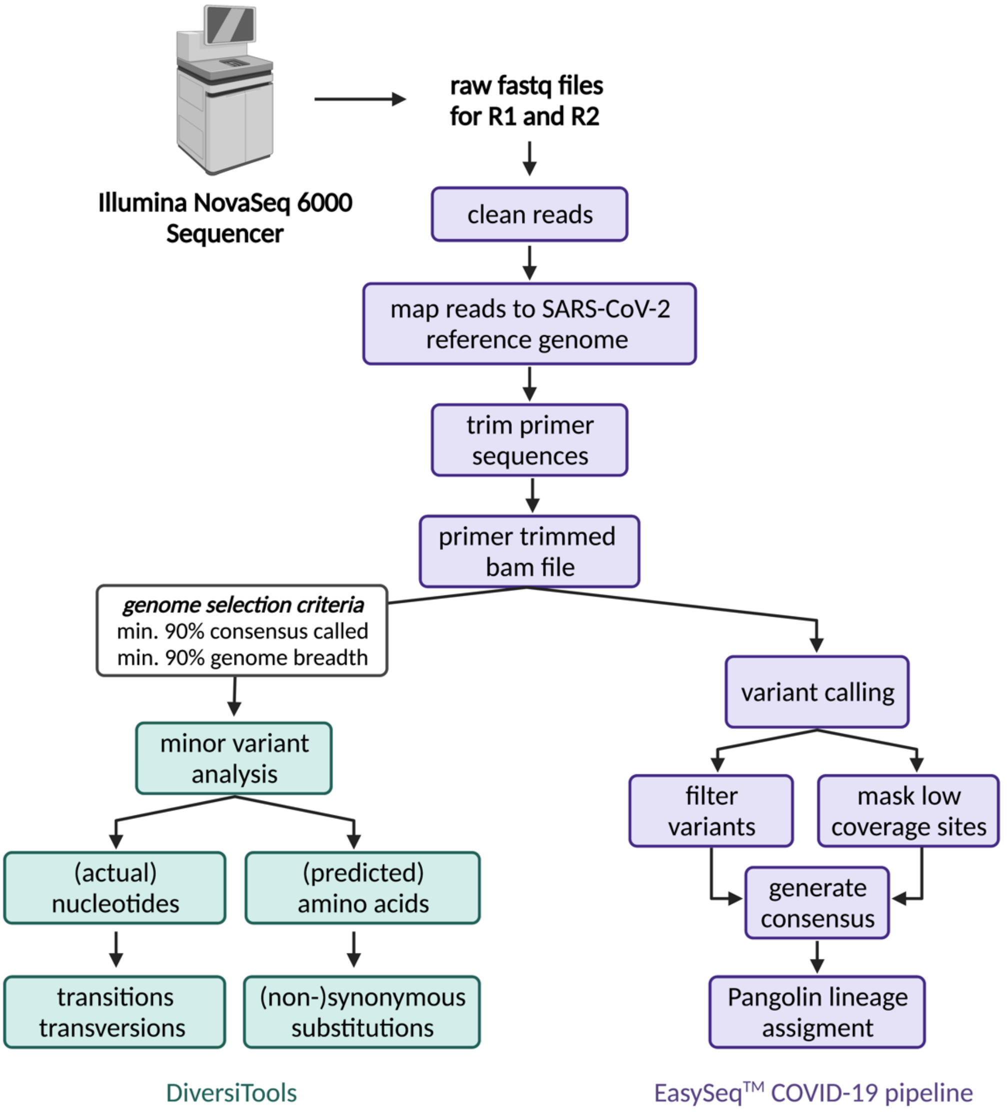

Supplementary Fig. 5: Computational workflow used to generate SARS-CoV-2 genomic data, assign PANGO lineage and analyse minor genomic variants. The first pipeline, EasySeq™ COVID-19 (purple, version 0.9) performs quality control steps, maps to the Wuhan-Hu-1 (NC045512.2) reference genome, variant calls and generates a consensus genome for each sample. Default parameters were used and are as follows: variant call threshold = 0.5; variant calling quality threshold = 20; variant calling minimum depth = 10. Pangolin (version 4.0.6) was used to assign SARS-CoV-2 lineage, with maximum ambiguity set at 0.3. Samples that pass the genome quality criteria are fed into the DiversiTools pipeline (green) which uses the primer-trimmed alignment file and its associated index file (produced in the EasySeq™ pipeline) along with the reference genome and a coding region file to analyse the minor genomic variation and predict the amino acid sequence based on the genomic data. Created with Biorender.com.
