# Supplementary material for: Characterisation of SARS-CoV-2 genomic variation in response to molnupiravir treatment in the AGILE Phase IIa clinical trial
Source: Nat Commun. 2022 Nov 26;13:7284. doi: 10.1038/s41467-022-34839-9 (PMC9701236; doi:10.1038/s41467-022-34839-9)
Supplement: Supplementary file 3 — Reporting Summary [file 41467_2022_34839_MOESM3_ESM.pdf]

nature portfolio

Corresponding author(s): Julian Hiscox and Thomas Fletcher

Last updated by author(s): Oct 31, 2022

Reporting Summary

Nature Portfolio wishes to improve the reproducibility of the work that we publish. This form provides structure for consistency and transparency in reporting. For further information on Nature Portfolio policies, see our Editorial Policies and the Editorial Policy Checklist.

Statistics

For all statistical analyses, confirm that the following items are present in the figure legend, table legend, main text, or Methods section.

n/a	Confirmed	
☐	☒	The exact sample size (*n*) for each experimental group/condition, given as a discrete number and unit of measurement
☐	☒	A statement on whether measurements were taken from distinct samples or whether the same sample was measured repeatedly
☐	☒	The statistical test(s) used AND whether they are one- or two-sided<br>*Only common tests should be described solely by name; describe more complex techniques in the Methods section.*
☒	☐	A description of all covariates tested
☐	☒	A description of any assumptions or corrections, such as tests of normality and adjustment for multiple comparisons
☒	☐	A full description of the statistical parameters including central tendency (e.g. means) or other basic estimates (e.g. regression coefficient) AND variation (e.g. standard deviation) or associated estimates of uncertainty (e.g. confidence intervals)
☐	☒	For null hypothesis testing, the test statistic (e.g. *F*, *t*, *r*) with confidence intervals, effect sizes, degrees of freedom and *P* value noted<br>*Give P values as exact values whenever suitable.*
☒	☐	For Bayesian analysis, information on the choice of priors and Markov chain Monte Carlo settings
☒	☐	For hierarchical and complex designs, identification of the appropriate level for tests and full reporting of outcomes
☒	☐	Estimates of effect sizes (e.g. Cohen's *d*, Pearson's *r*), indicating how they were calculated

Our web collection on statistics for biologists contains articles on many of the points above.

Software and code

Policy information about availability of computer code

Data collection	Raw fastq files were processed using the EasySeq_covid19 (version 0.9, code available at https:/ /github.com/JordyCoolen/easyseq_covid19), with sequenced mapped using the Wuhan-Hu-1 reference genome (NC045512.2 https://www.ncbi.nlm.nih.gov/nuccore/NC_045512.2).
Data analysis	SARS-CoV-2 lineages were assigned using Pangolin version 4.0.6 (available at https://github.com/cov-lineages/pangolin). DiversiTools was used to analyse the the minor variants of each sample (available at https://github.com/josephhughes/DiversiTools). RStudio version 4.0.2 was used to visualise the DiversiTools output data, using the tidyverse package (version 1.3.2) for data manipulation. Statistical tests were conducted using the Rstatix package (version 0.7.0). All graphs were created using ggplot2 (version 3.3.6). Figures were compiled using cowplot (version 1.1.1) and magick (version 2.7.3) packages. Custom R scripts developed for this study are available at https://github.com/ Hiscox-lab/AGILE-molnupiravir-viral-genomics.

For manuscripts utilizing custom algorithms or software that are central to the research but not yet described in published literature, software must be made available to editors and reviewers. We strongly encourage code deposition in a community repository (e.g. GitHub). See the Nature Portfolio guidelines for submitting code & software for further information.

March 2021

Data

Policy information about availability of data

All manuscripts must include a data availability statement. This statement should provide the following information, where applicable:

- Accession codes, unique identifiers, or web links for publicly available datasets
- A description of any restrictions on data availability
- For clinical datasets or third party data, please ensure that the statement adheres to our policy

All raw sequencing data used in the analysis have been deposited to the National Center for Biotechnology Information (NCBI) Short Read Archive (SRA) under accession code PRJNA854613 available at https://www.ncbi.nlm.nih.gov/bioproject/PRJNA854613. Source data has been included and details 1. Genomic analysis metadata; 2. The exact p-values for Figs. 1c, 1d and Supplementary Fig. 1; 3. Accession numbers for all the raw sequence files deposited in the SRA repository PRJNA854613; and 4. The trial participants' co-variate information.

Human research participants

Policy information about studies involving human research participants and Sex and Gender in Research.

Reporting on sex and gender	Gender information was not collected as part of this study.
Population characteristics	There were 103 females and 77 males recruited to the study. There were 90 vaccinated and 90 un-vaccinated participants inn the study. The age of the participants ranged from 18 to 81, median age 43. 151 participants identified as "White English, Welsh, Scottish, Northern Irish, or British"; 20 as "Any other White background"; 2 as "Asian or British Asian: Indian"; and 1 each as "Mixed or part of multiple ethnic groups: White and Black African", "Mixed or part of multiple ethnic groups: White and Asian", "Black, African, Caribbean, or Black British–Caribbean", "Asian or British Asian: Pakistani", "Asian or British Asian: Chinese", "Any other Black, African, or Caribbean background" and "Any other Asian background". All information can be also found in the Source File.
Recruitment	Participants were recruited across five UK National Institute for Health and Care Research (NIHR) Clinical Research Facility sites (in Liverpool, Manchester, Lancashire, Southampton, and London). Sites would recruit using any of the positive patient lists they had access to, trial posters at hospital sites and adverts on hospital websites. The most successful method being the positive patient lists. The lists ranged from local positive hospital lists, which would include staff, to local government pillar II positive lists. Research staff at each site would call potential participants from the positive lists and if interested would check basic eligibility. The potential participant would then be brought in for a screening.
Ethics oversight	All participants provided written informed consent before enrolment. The study protocol was reviewed and approved by the UK Medicines and Healthcare product Regulatory Agency (MHRA) (EudraCT 2020-001860-27) and West Midlands Edgbaston Research Ethics Committee (20/WM/0136).

Note that full information on the approval of the study protocol must also be provided in the manuscript.

Field-specific reporting

Please select the one below that is the best fit for your research. If you are not sure, read the appropriate sections before making your selection.

☒ Life sciences ☐ Behavioural & social sciences ☐ Ecological, evolutionary & environmental sciences

For a reference copy of the document with all sections, see nature.com/documents/nr-reporting-summary-flat.pdf

Life sciences study design

All studies must disclose on these points even when the disclosure is negative.

Sample size	Sample size was determined by availability of participants enrolled in the AGILE CST-2 study, and sequence quality determination of the SARS-CoV-2 RNA derived from the nasopharyngeal swabs obtained throughout the trial.
Data exclusions	Participants were excluded from the minor variant analysis if all three of their samples met the following criteria: 1) the dominant genome sequence had a minimum 90% consensus called and 2) 90% of genome positions had a minimum coverage of 200X.
Replication	The sequencing of the RNA samples was not repeated due to limited sample availability. For comparisons made between the treatment allocations, each individual in a group represented a repeat for the condition (i.e. 90 molnupiravir treated participants vs 90 placebo treated participants).
Randomization	Using a permuted block (block size 2 or 4) method and stratifying by site, participants were randomly assigned (1:1) to receive either molnupiravir plus standard of care or placebo plus standard of care. The randomisation sequence was generated by use of STATA (version 16) by an independent statistician (who had no further involvement in the trial) and used to prepare labelled placebo and treatment packs, which were assigned sequentially to patients on randomisation. Placebo and molnupiravir were provided in tablets of identical appearance.

Participants, the staff giving and assessing the interventions, and those who analysed the data were masked to treatment allocation until the end of the study.

Blinding	Investigators in this study were blinded as per the AGILE CST-2 study protocol. Sequencing data analysis was partially performed during this blinding period, with mitigations in place (samples separated into two arms, but not identified).

Reporting for specific materials, systems and methods

We require information from authors about some types of materials, experimental systems and methods used in many studies. Here, indicate whether each material, system or method listed is relevant to your study. If you are not sure if a list item applies to your research, read the appropriate section before selecting a response.

Materials & experimental systems

n/a	Involved in the study	
☒	☐	Antibodies
☒	☐	Eukaryotic cell lines
☒	☐	Palaeontology and archaeology
☒	☐	Animals and other organisms
☐	☒	Clinical data
☒	☐	Dual use research of concern

Methods

n/a	Involved in the study	
☒	☐	ChIP-seq
☒	☐	Flow cytometry
☒	☐	MRI-based neuroimaging

Clinical data

Policy information about clinical studies

All manuscripts should comply with the ICMJEguidelines for publication of clinical research and a completedCONSORT checklist must be included with all submissions.

Clinical trial registration	NCT04746183
Study protocol	Study protocol for the AGILE platform is available at https://trialsjournal.biomedcentral.com/articles/10.1186/s13063-021-05458-4
Data collection	The clinical study was conducted at five UK National Institute for Health and Care Research (NIHR) Clinical Research Facility (CRF) sites (in Liverpool, Manchester, Lancashire, Southampton and London), coordinated by the NIHR Southampton Clinical Trials Unit and sponsored by the University of Liverpool. Recruitment was from November 2020 to March 2022.
Outcomes	For the secondary virological study described in this article, no outcomes were predefined. The hypothesis tested was that treatment of patients with molnupiravir would result in the detectable increase in transition mutations over time, as previously described in vitro and in animal models.
